# Evaluation of a new multipurpose whole-body CzT-based camera: comparison with a dual-head Anger camera and first clinical images

**DOI:** 10.1186/s40658-020-0284-5

**Published:** 2020-03-17

**Authors:** Cédric Desmonts, Mohammed Abdeldjalil Bouthiba, Blandine Enilorac, Catherine Nganoa, Denis Agostini, Nicolas Aide

**Affiliations:** 1 Department of Nuclear Medicine, Caen University Hospital, Avenue de la côte de Nacre, 14033 Caen Cedex 9, France; 2 Normandie University, Caen, France; 3 Inserm Anticipe, Normandie University, Caen, France

**Keywords:** Molecular imaging, Multipurpose CzT-camera, SPECT, Whole-body imaging

## Abstract

**Background:**

Evaluate the physical performance of the VERITON CzT camera (Spectrum Dynamics, Caesarea, Israel) that benefits from new detection architecture enabling whole-body imaging compared to that of a conventional dual-head Anger camera.

**Methods:**

Different line sources and phantom measurements were performed on each system to evaluate spatial resolution, sensitivity, energy resolution and image quality with acquisition and reconstruction parameters similar to those used in clinical routine. Extrinsic resolution was assessed using ^99m^Tc capillary sources placed successively in air, in a head and in a body phantom filled with background activity. Spectral acquisitions for various radioelements used in nuclear medicine (^99m^Tc, ^123^I, ^201^Tl, ^111^In) were performed to evaluate energy resolution by computing the FWHM of the measured photoelectric peak. Tomographic sensitivity was calculated by recording the total number of counts detected during tomographic acquisition for a set of source geometries representative of different clinical situations. Sensitivity was also evaluated in focus mode for the CzT camera, which consisted of forcing detectors to collect data in a reduced field-of-view. Image quality was assessed with a Jaszczak phantom filled with 350 MBq of ^99m^Tc and scanned on each system with 30-,20-,10- and 5-min acquisition times.

**Results:**

Extrinsic and tomographic resolution in the brain and body phantoms at the centre of the FOV was estimated at 3.55, 7.72 and 6.66 mm for the CzT system and 2.47, 7.75 and 7.72 mm for the conventional system, respectively. The energy resolution measured at 140 keV was 5.46% versus 9.21% for the Anger camera and was higher in a same manner for all energy peaks tested. Tomographic sensitivity for a point source in air was estimated at 236 counts·s^−1^·MBq^−1^ and increased to 1159 counts·s^−1^·MBq^−1^ using focus mode, which was 1.6 times and 8 times greater than the sensitivity measured on the scintillation camera (144 counts·s^−1^·MBq^−1^). Head and body measurements also showed higher sensitivity for the CzT camera in particular with focus mode. The Jaszczak phantom showed high image contrast uniformity and a high signal-to-noise ratio on the CzT system, even when decreasing acquisition time by 6-fold. Representative clinical cases are shown to illustrate these results.

**Conclusion:**

The CzT camera has a superior sensitivity, higher energy resolution and better image contrast than the conventional SPECT camera, whereas spatial resolution remains similar. Introduction of this new technology may change current practices in nuclear medicine such as decreasing acquisition time and activity injected to patient.

## Background

Dedicated cardiac CzT-based cameras have been commercially available for a decade. These cameras have a higher sensitivity and better energy resolution than Anger cameras [1–4]. Gains in sensitivity have drastically changed patient management of myocardial SPECT perfusion exploration in particular, with a decrease in acquisition time or total activity administered to the patient [5, 6]. Moreover, the better discrimination of radioisotope photopeaks opens new perspectives with dual-isotope acquisitions [7–9]. Most recently, new cameras equipped with wide-field CzT detectors have been introduced to extend the use of this technology to all nuclear medicine explorations. GE Healthcare was the first manufacturer to commercialize such a multi-purpose CzT camera [10]. This camera is equipped similarly to an Anger camera with two large, flat detectors, thus keeping the same detection geometry as conventional SPECT systems. More recently, Spectrum Dynamics has unveiled a new camera architecture with a ring-configuration CzT detector. The latter has been previously described by Goshen et al. [11].

The purpose of the present study was to evaluate the SPECT performance of the Veriton CzT camera and to compare it with a conventional scintillation camera using the recommended parameters for clinical routine.

## Methods

### CzT camera

**Fig. 1:**
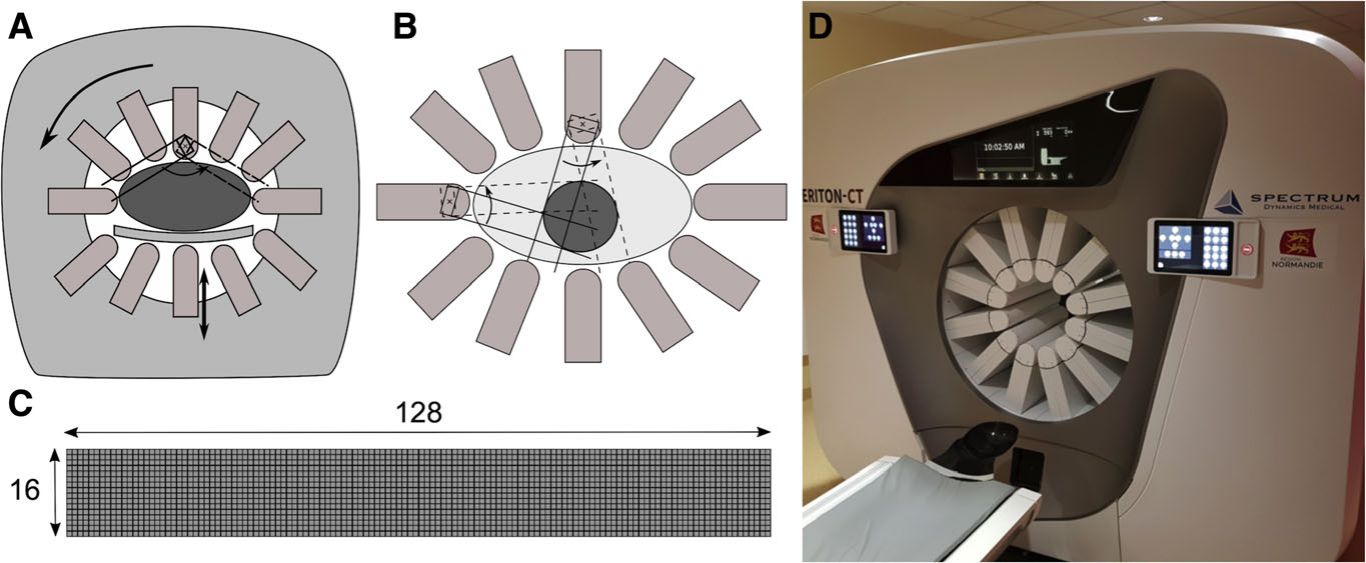
General camera architecture showing the different movements of the 12 detectors (**a**), schematic principle of the focus mode showing the reduced swipe motion of detectors to a predefined region of interest shown in dark grey (**b**), design of the detection column consisting of an array of 16 by 128 pixel units (**c**) and picture of the system (**d**)

All tests were performed on the first VERITON camera (Spectrum Dynamics, Caesarea, Israel) commercially available (FDA and EU clearance was obtained for the present study), installed at the University Hospital of Caen, France. The camera consisted of 12 columns of detectors arranged in a ring configuration. Each column was composed of a 16 × 128 array of CzT pixel units and equipped with high sensitivity tungsten parallel-hole collimators in alignment with the pixel array. Detector surfaces were equipped with skin sensors to get as close to the body contours as possible during acquisition. Acquisition could be performed either in manual mode with a circular field of view or in body-contouring mode. Acquisition consisted of a few angular acquisition steps ensured by a gantry rotation. The number of required steps, typically four for normal body contours, was dependent upon the size of the field of view. At each acquisition step, a sweeping motion of each column of detectors was performed to collect data from the entire field of view. A focus mode was also available to force detector movement to collect data in a region of interest defined by the user on a pre-scan acquisition. Figure 1 describes the general camera architecture and explains the focus mode. In this study, all acquisitions were performed with a default energy window set by manufacturer at 20% centred on the photopeak of ^99m^Tc and a matrix size of 256 × 256, resulting in a squared pixel size of 2.46 mm. Images were reconstructed with a proprietary OSEM algorithm including the use of an internal pre-iteration median convolution kernel for noise suppression, with 10 iterations, 32 subsets and without the use of a post-reconstruction filter.

### Anger camera

The Anger camera used in this study was a dual-head Symbia camera (Siemens, Erlangen, Germany) equipped with a 3/8 inch NaI (Tl) crystal. Detectors were equipped with a low-energy, high-resolution (LEHR) collimator. A full calibration of the detector was done before the evaluation, and the camera passed all calibration tests. SPECT acquisitions were performed with standard reconstruction parameters used in clinical routine as recommended by the manufacturer. With the exception of some tests that needed default parameters to be adjusted, all acquisitions were performed with the following parameters: an energy window of 15% centred at ^99m^Tc photopeak (140 keV), 64 projections with a matrix size of 128x128, resulting in a 4.8-mm-square pixel size. Reconstructions were performed with a 2D-OSEM algorithm with 8 iterations and 4 subsets and without post-filtering.

### Patient examinations

As part of the FDA clearance scheme, several patients were imaged within the framework of a phase I study approved by the regional Ethics committee under the number ID RCB: 2017-A01448-45, registered at clinicaltrials.gov with the following number: NCT03438123. We selected cases representative of various isotopes, organ sizes and shapes, as well as count statistics.

### Extrinsic resolution

Extrinsic resolution was assessed using 1-mm-diameter capillaries (PTW Freiburg) placed in air and filled with approximately 20 MBq/mL of ^99m^Tc. The three-line sources were positioned parallel to the longitudinal axis of rotation, 0, 4.5 and 9 cm from the centre of the field of view. Five-minute tomographic acquisitions were performed on both cameras with a circular shape, a radius of rotation of 15 cm and a matrix size 256 × 256 to obtain the finest linear sampling. The resulting pixel size was 2.46 mm for the CzT camera and 2.40 mm for the Anger camera. Acquisitions were performed on the Anger camera with LEHR collimators. Different sets of images were reconstructed using an OSEM reconstruction algorithm by varying the total number of iterations from 8 to 640, except for the Anger camera for which the maximum number of iterations was limited to 320, and without post-reconstruction filters applied. Profiles passing through the maximum pixel value of each source were drawn in radial and tangential directions. A Gaussian function was fitted to compute the full width at half maximum (FWHM) of each profile measurement.

### Tomographic resolution

As final image resolution depends on both acquisition and reconstruction parameters, tomographic resolution was estimated with phantoms simulating different clinical situations used in routine.

To mimic a clinical brain acquisition, tomographic resolution was first estimated using a head phantom (from International Electrotechnical Commission (IEC) Standard 61675-2) equipped with three cavities located in the middle and at 4.5 and 9 cm from the centre. The same line sources used for extrinsic resolution measurement were introduced in the phantom filled with a background activity of 56 MBq of ^99m^Tc. The phantom was positioned at the centre of the camera, and acquisitions were performed with a circular radius of rotation of 15 cm. For the CzT camera, a 640-s acquisition was performed with a matrix size of 256 × 256, and for the anger camera, 64 projections of 10 s per head were performed with a matrix size of 128 × 128 and a zoom factor 1.45.

To mimic a clinical body acquisition, the same experiment was then carried out using a body phantom (from IEC standard 61675-1) filled with a 97-MBq background activity of ^99m^Tc using the previous line sources placed at the same distance from the centre of the phantom. Acquisitions were performed on both cameras with the phantom placed at the centre of field of view in autocontour mode. For the CzT camera, a 640 s was performed with a matrix size of 256 × 256, and for the anger camera, 32 projections per head of 20 s were performed with a matrix size of 128 × 128 without zoom factor. All acquisitions were reconstructed by varying the total number of iterations from 8 to 640, except for the Anger camera for which the matrix size used limited the maximum number of iterations to 320 for the head phantom and 160 for the body phantom, and by applying a Gaussian post-reconstruction filter of 5-mm FHHM. A profile passing through the maximum pixel value of the sources was drawn. A Gaussian function was fitted to determine the FWHM of each line source in the radial and tangential directions.

### Energy resolution

Energy resolution was measured for the most commonly used radioelements in nuclear medicine: ^201^Tl (70 keV, 167 keV), ^99m^Tc (140 keV), ^123^I (159 keV) and ^111^In (171 keV, 245 keV). Source preparation and spectral acquisition were carried out according to manufacturer’s recommendations. For the CzT camera, energy spectra were acquired using fillable cylinders of around 42 cm-length and 0.9 cm of inner diameter, filled with a solution of 110 to 370 MBq depending on the tested radioisotope and placed at the centre of the field of view. On the Anger camera, point sources were used instead with an activity adjusted so that the count rate measured on the camera was between 15 and 50 kilocounts per second at acquisition start and centred in the field of view equidistant from the 2 detectors equipped with LEHR collimators. Energy spectra were acquired for all pixel elements on the CzT camera and for the two detectors on the Anger camera. Spectral acquisition was stopped when the number of counts per pixel reached 25 kilocounts for the CzT camera, and when the number of counts recorded at the maximum energy peak was about 32 kilocounts for the Anger camera. Each peak of energy was fitted by a Gaussian function to calculate FWHM. The resulting energy resolution was expressed as the mean FWHM for all pixel units +/− Standard Deviation (SD) for the CzT camera and as the mean FWHM for the two detectors +/− SD for the Anger camera.

### Tomographic sensitivity

The purpose of this test was to assess tomographic sensitivity under conditions used in clinical routine rather than to evaluate the intrinsic sensitivity of the system. Therefore, the default parameters used in clinical routine were selected. As the global sensitivity of the system depends both on the nature of the detectors and on the geometry of detection, sensitivity was assessed for different types of radiation sources, including conditions representative of clinical situations. Sensitivity was first evaluated on each camera with a point source of approximately 25 MBq of ^99m^Tc placed at the centre of the field of view and a radius of rotation of 15 cm. Measures were successively performed with the source placed in air, at the centre of a water-filled head phantom and at the centre of a water-filled body phantom (phantoms are those described in the tomographic resolution section). Two different count measurements were performed on the CzT camera: one on the entire field of view and a second with a focus of the detector on a circular region of interest centred on the point source. Sensitivity was then evaluated with the head and the body phantoms filled with a uniform solution of approximately 350 MBq of ^99m^Tc and placed at the centre of the field of view. Acquisition was performed with a radius of rotation of 15 cm for the head phantom and in autocontour mode for the body phantom. The exact activity of the prepared sources was assessed by applying radioactive decay correction to the measurement performed with a calibrated well counter (MEDI-405, Medisystem, Guyancourt, France). The total number of counts was recorded for each acquisition of 450-s duration, and the sensitivity was calculated as follows:

$$ \mathrm{Sensitivity}\kern0.35em \left(\mathrm{counts}.{\mathrm{MBq}}^{-1}.{s}^{-1}\right)=\frac{\mathrm{total}\kern0.35em \mathrm{recording}\kern0.35em \mathrm{counts}}{\mathrm{acquisition}\kern0.35em \mathrm{time}\ (s)\times \mathrm{source}\kern0.35em \mathrm{activity}\ \left(\mathrm{MBq}\right)} $$

### Image quality

A Flangeless Deluxe Jaszczak phantom filled with 350 MBq of ^99m^Tc was scanned on each camera to evaluate global image quality, lesion detectability and uniformity of the reconstructed slice. Phantom was positioned on a headrest at the centre of the field of view, and acquisitions were performed in autocontour mode with the default parameters described above. Focus mode was not activated on CzT camera. Images were acquired on Anger camera with a zoom factor of 1.45 resulting in a squared pixel size of 3.3 mm. To evaluate the influence of acquisition time, four acquisitions were tested as follows: 30, 20, 10 and 5 min. Images were corrected for attenuation with the Chang attenuation method, applying a coefficient of attenuation of 0.11 cm^-1^. For each acquisition, the total number of counts was recorded. To easily compare the SPECT performance of the two cameras, image quality was quantitatively assessed by computing contrast, uniformity and noise index values, as proposed by the American Association of Physicists in Medicine [12]. All regions of interest and statistical tests were computed using the AMIDE software [13].

### Image contrast

To calculate the image contrast for each dataset, the transverse slice where the cold spheres were the most visible was selected. The minimum pixel counts were determined for each spheres in the chosen slice, and the mean pixel value was calculated in a 15 × 15-pixel square ROI drawn in the uniform background of the phantom. The image contrast was then calculated for each cold sphere as follows:

$$ \mathrm{Contrast}=\frac{\mathrm{mean}\kern0.35em {\mathrm{pixel}}_{\mathrm{background}}-\min \kern0.35em {\mathrm{pixel}}_{\mathrm{coldsphere}}}{\mathrm{mean}\kern0.35em {\mathrm{pixel}}_{\mathrm{background}}} $$

### Tomographic uniformity

To calculate the image uniformity, a 15 × 15-pixel square ROI was drawn on a transaxial slice located on the uniform part of the phantom. The mean, maximum, minimum and standard deviations of the pixel values within the ROI were recorded. Integral uniformity and root mean square noise (RMS noise) were calculated with the following formulas:

$$ \mathrm{Integral}\kern0.35em \mathrm{uniformity}\kern0.35em \left(\%\right)=\frac{\left(\max \kern0.35em \mathrm{pixel}-\min \kern0.35em \mathrm{pixel}\right)}{\max \kern0.35em \mathrm{pixel}+\min \kern0.35em \mathrm{pixel}}\times 100 $$

$$ \mathrm{RMS}\kern0.35em \mathrm{noise}\left(\%\right)=\frac{\mathrm{standard}\kern0.35em \mathrm{deviation}}{\mathrm{mean}\kern0.35em \mathrm{pixel}\kern0.35em \mathrm{value}}\times 100 $$

### Clinical images

Four clinical cases were studied to compare image quality obtained on the CzT and Anger cameras: a ^99m^Tc-pertechnetate thyroid scan (15 min after injection of 117 MBq, 10 min scan duration), a ^99m^Tc-HMDP bone scintigraphy (3 h after injection of 643 MBq, 10-min scan duration), a ^99m^Tc-DMSA renal scan (4-h after injection of 111 MBq, 10-min scan duration ) and a prostate cancer treatment imaging study after injection of ^223^Ra (48-h after injection of 5.27 MBq, 30-min scan duration). Patients were scanned successively on each camera with the same acquisition time. The patients were first scanned on the Anger camera and then on the CzT camera with correction of scan duration for activity decay. To quantitatively assess image quality, the contrast-to-noise ratio (CNR) was computed for each lesion detected. Two spherical volumes of interest (VOI) of 5-mm diameter were manually drawn on clinical images using the AMIDE software [13]: the first one centred on the lesion and the second on healthy tissue near the lesion. CNR was calculated as follows:

$$ \mathrm{CNR}=\frac{\mathrm{mean}\ {\mathrm{pixel}\ \mathrm{value}}_{\mathrm{lesion}}-\mathrm{mean}\ {\mathrm{pixel}\ \mathrm{value}}_{\mathrm{healthy}\ \mathrm{tissue}}\ }{{\mathrm{standard}\ \mathrm{deviation}}_{\mathrm{healthy}\ \mathrm{tissue}}} $$

All images were analysed, and VOIs were manually drawn jointly by a nuclear medicine physician and an expert in medical physics with more than 10 years of experience.

## Results

### Extrinsic resolution

**Fig. 2:**
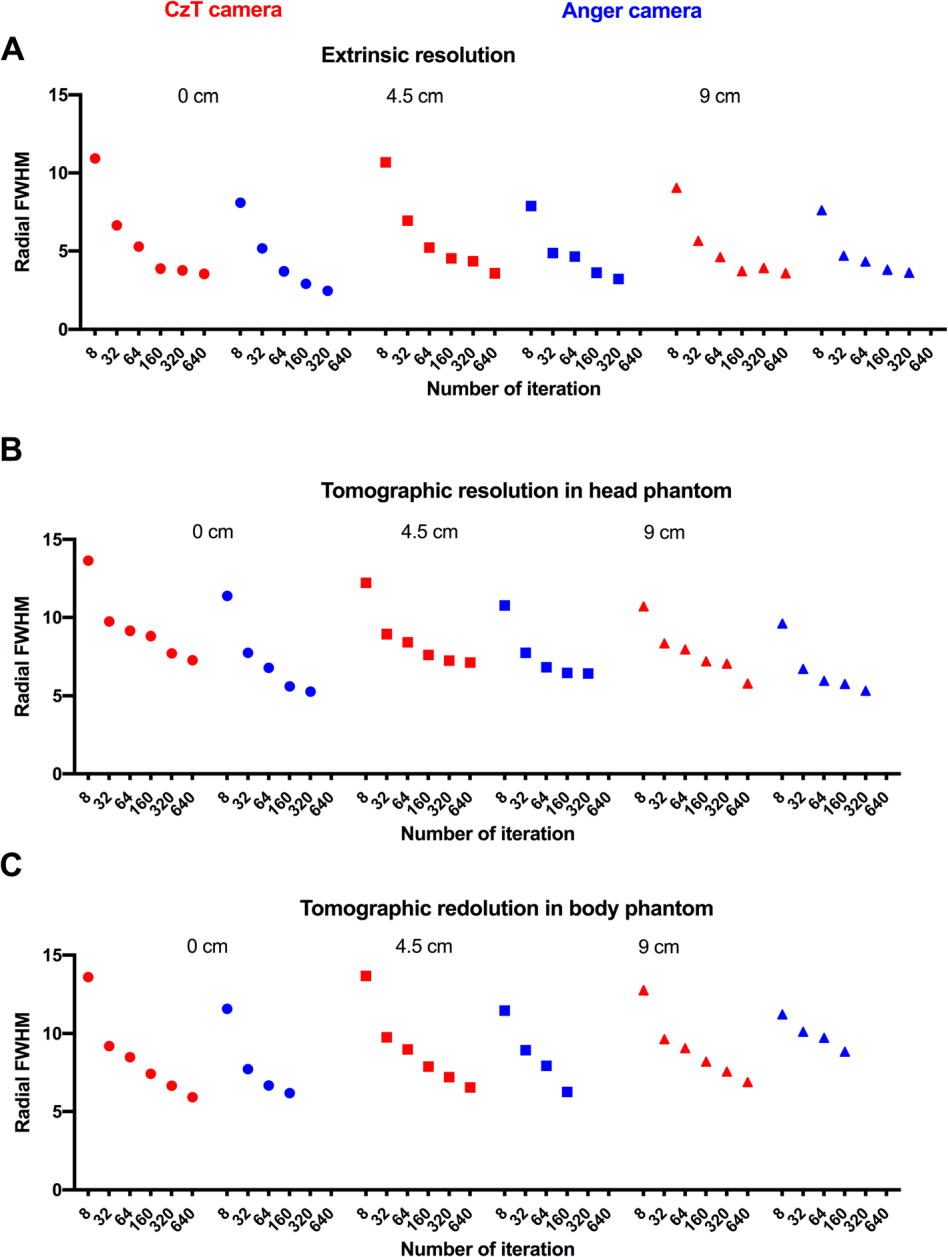
Estimated radial spatial resolution of Anger and CzT cameras as a function of the distance to the centre of the field of view (ranging from 0 to 9 cm) measured in air (**a**) in a head phantom (from IEC Standard 61675-2) (**b**) and in a body phantom (from IEC Standard 61675-1) (**c**)

Figure 2 a presents the measured FWHM as a function of number of iterations, at 0, 4.5 and 9 cm from the centre of the field of view for both cameras.

The maximum FWHM obtained at the convergence of the reconstruction algorithm on the CzT camera for the three-line sources placed at 0, 4.5 and 9 cm from the centre of the camera was 3.54, 3.58 and 3.59 mm, respectively, in the radial direction. The respective results obtained on the Anger camera were 2.47, 3.24 and 3.63 mm in the radial direction. While the measured extrinsic resolution was slightly higher for the Anger camera at the centre of the field of view, the resolution decreased when approaching the surface of detectors while it remained constant with the CzT camera whatever the source position.

### Tomographic resolution

Figure 2 b and c present the measured FWHM as a function of the number of iterations for the brain and body phantoms at 0, 4.5 and 9 cm from the centre of the field of view for both cameras.

For the brain phantom, using the standard reconstruction settings, the calculated FWHM obtained on the CzT camera with 10 iterations and 32 subsets for the three-line sources placed at 0, 4.5 and 9 cm from the centre of the camera were 7.72, 7.26 and 7.07 mm in the radial direction. The respective results obtained for the Anger camera with 4 iterations and 8 subsets were 7.75, 7.76 and 6.73 mm.

For the body phantom, using the standard reconstruction settings, the calculated FWHM obtained on the CzT camera for the three-line sources placed at 0, 4.5 and 9 cm from the centre of the camera were 5.92, 6.55 and 6.89 mm in the radial direction. The respective results obtained for the Anger camera were 7.72, 8.93 and 10.12 mm.

### Energy resolution

Table 1 shows the energy resolution estimated for the photoelectric peaks of the four tested radioisotopes. Energy resolution was expressed as the measured FWHM normalized by the theoretical energy of the photoelectric peak. Estimated energy resolution was superior for the CzT camera at each energy level, with a ratio in a range of 1.68 to 2.55 compared to the Anger camera.

**Table 1:** Percent FWHM measured on CzT and Anger cameras for 6 energy peaks. Results are expressed in mean (SD)

Radioisotope	^201^Tl		^99m^Tc	^123^I	^111^In	
Energy peak (keV)	70	167	140	159	171	245
% FWHM Anger camera	14.08 (0.19)	11.98 (1.66)	9.21 (0.07)	10.24 (0.79)	10.78 (0.03)	9.03 (0.35)
% FWHM CzT camera	5.77 (1.30)	4.67 (1.13)	5.46 (0.59)	5.33 (0.62)	6.27 (1.08)	4.5 (0.75)

### Tomographic sensitivity

Table 2 shows the measured sensitivity obtained on both cameras for the different tested source geometries. For the point source placed in air, the recorded sensitivity was 1.6 times higher on the CzT camera than for the Anger camera and 8 times higher if the focus mode was activated. With the same source placed successively at the centre of a cold water-filled head and body phantom, sensitivities were respectively 1.28 and 1.08 times higher on the CzT camera and 6.01 and 5.75 times higher with the focus mode activated. For uniformly filled phantoms with radioactive solution, measured sensitivities were 1.5 and 1.25 times higher on the CzT camera respectively for the head and body phantoms.

**Table 2:** Sensitivity measured with different source geometries on Anger and CzT cameras with and without focus mode

Sensitivity in counts·s^−1^·MBq^−1^	Anger camera	CzT camera without focus	CzT camera with focus
Point source in air	143.61	236.68	1159.33
Point source in head phantom	56.97	73.42	342.40
Point source in body phantom	35.94	39.05	207.70
Head phantom	72.69	107.18	na
Body phantom	52.75	65.79	na

### Image quality

**Fig. 3:**
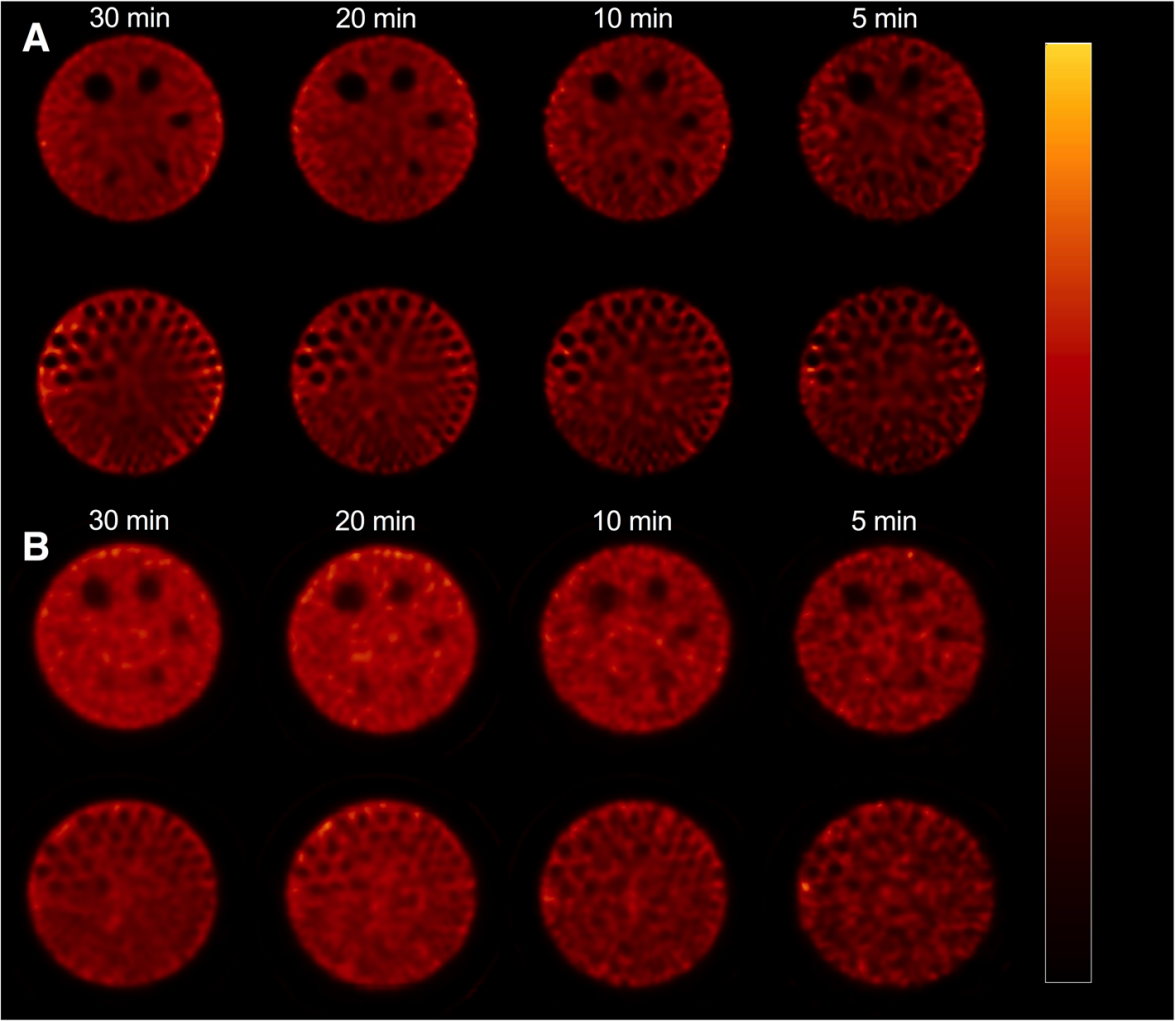
Axial reconstructed slices (similar scale with min and max threshold respectively set to 0 and 125%) of the Jaszczak phantom obtained for CzT (**a**) and Anger cameras (**b**) centred on cold spheres (top rows) or capillaries (bottom rows) for 30, 20, 10 or 5 min of acquisition time (left to right)

Figure 3 shows the different parts of the Jaszczak phantom, rod sections and cold spheres, for the four tested acquisition times and for both cameras. For the 30-min acquisition performed on the CzT camera, the first five cold spheres, corresponding to the spheres with an inner diameter of 31.8, 25.4, 19.1, 15.9 and 12.7 mm, were clearly visible, with a good image contrast. The five largest sections of rods, corresponding to the rods with diameter of 12.7, 11.1, 9.5, 7.9 and 6.4 mm, were entirely to partially visible but showed a significant decrease in sharpness with depth in the last two sections of rods. Although the number of visible elements remained approximately the same when decreasing acquisition time, we noticed a decrease in global image quality, for the shortest acquisition time in particular, due to a significant increase in noise level. For the 30-min acquisition performed on the Anger camera, five cold spheres were visible, and only three rod sections were partially visible, with a relatively low image contrast compared to that observed with the CzT camera. We also noticed a rapid decrease in the number of visible elements when the acquisition time decreased.

### Image contrast

**Fig. 4:**
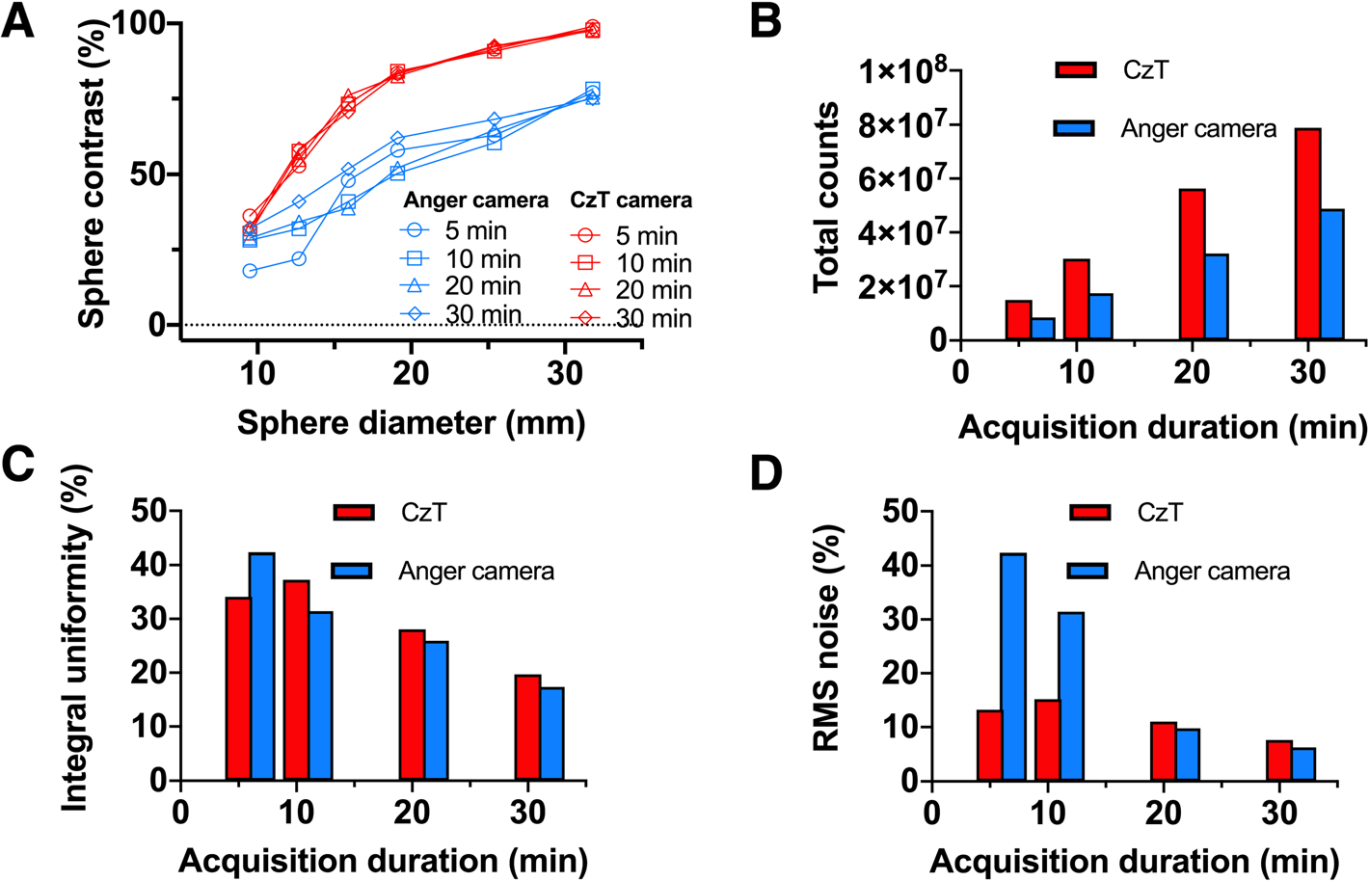
Image contrast measured as a function of sphere diameter at different acquisition times for CzT and Anger cameras (**a**). Total recorded counts (**b**), integral uniformity (**c**), and RMS noise (**d**) calculated for different acquisition times for CzT and Anger cameras

Figure 4 a shows the calculated image contrast as a function of sphere diameter for each acquisition time and both cameras. Contrast was far superior for the CzT camera for each sphere diameter, regardless of the acquisition time. Moreover, the contrast measured for a 5-min acquisition on the CzT camera was higher than that measured for a 30-min scan time on the Anger camera.

### Tomographic uniformity

Figure 4 b–d present the number of total counts recorded; the calculated integral uniformity and RMS noise for each acquisition performed, respectively. The total recorded counts confirmed the previous sensitivity measurements, with a higher count statistic obtained for the CzT camera than for the Anger camera. This gain in count statistic was combined with a better uniformity and a lower noise level measured in the reconstructed slices for the two shortest acquisition times compared to the Anger camera. In particular, the noise remained relatively constant for the CzT camera when the acquisition time was reduced, while it increased drastically on the Anger camera.

### Clinical images

**Fig. 5:**
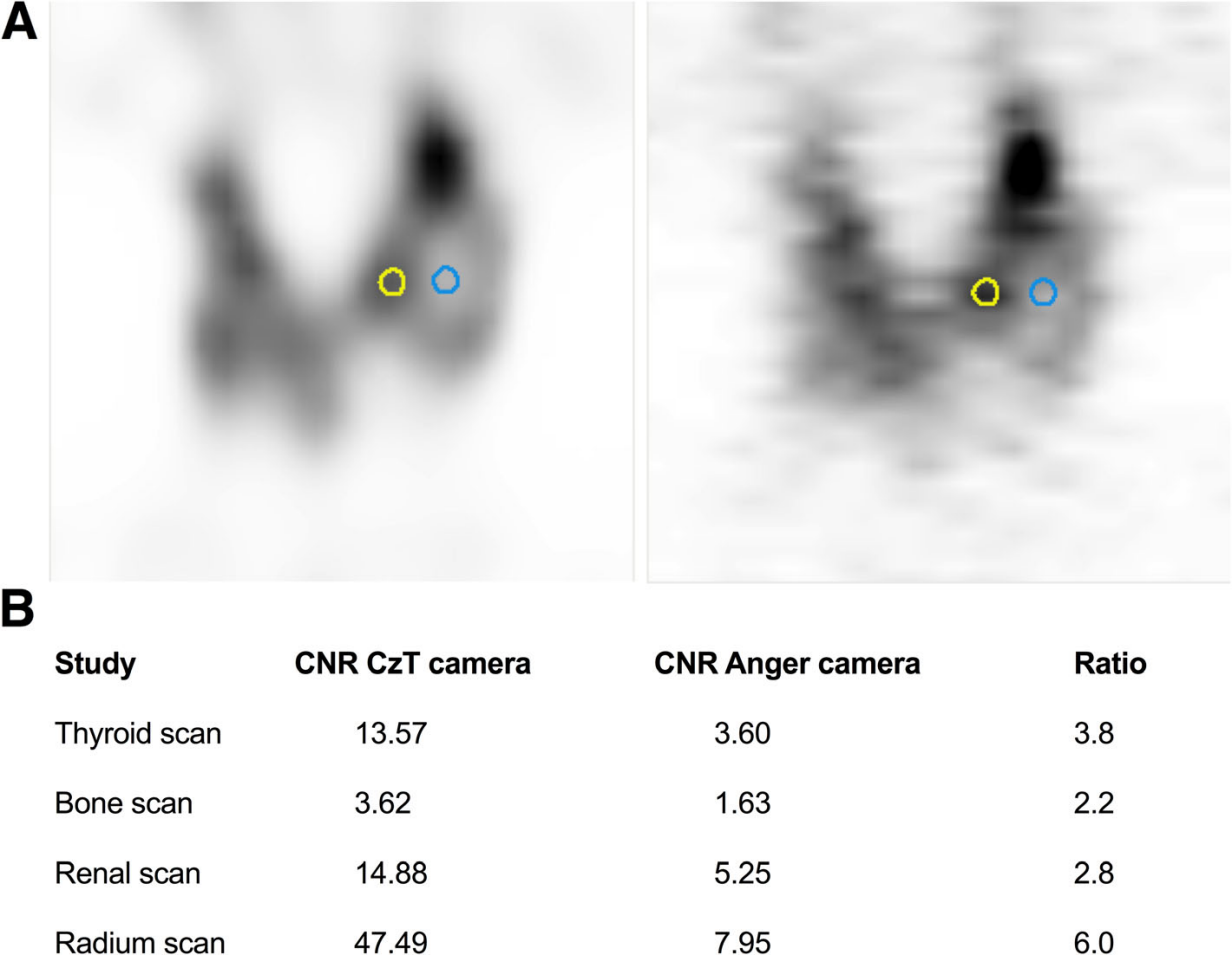
Example of a manually drawn VOIs for the thyroid study (**a**): spherical VOIs of 5 mm of diameter were drawn on lesion corresponding to a cold nodule (blue) and normal tissue (yellow) VOIs were drawn on CzT camera images (left) and copied to Anger camera images (right). The resulting CNRs were computed for each clinical case and both cameras (**b**)

**Fig. 6:**
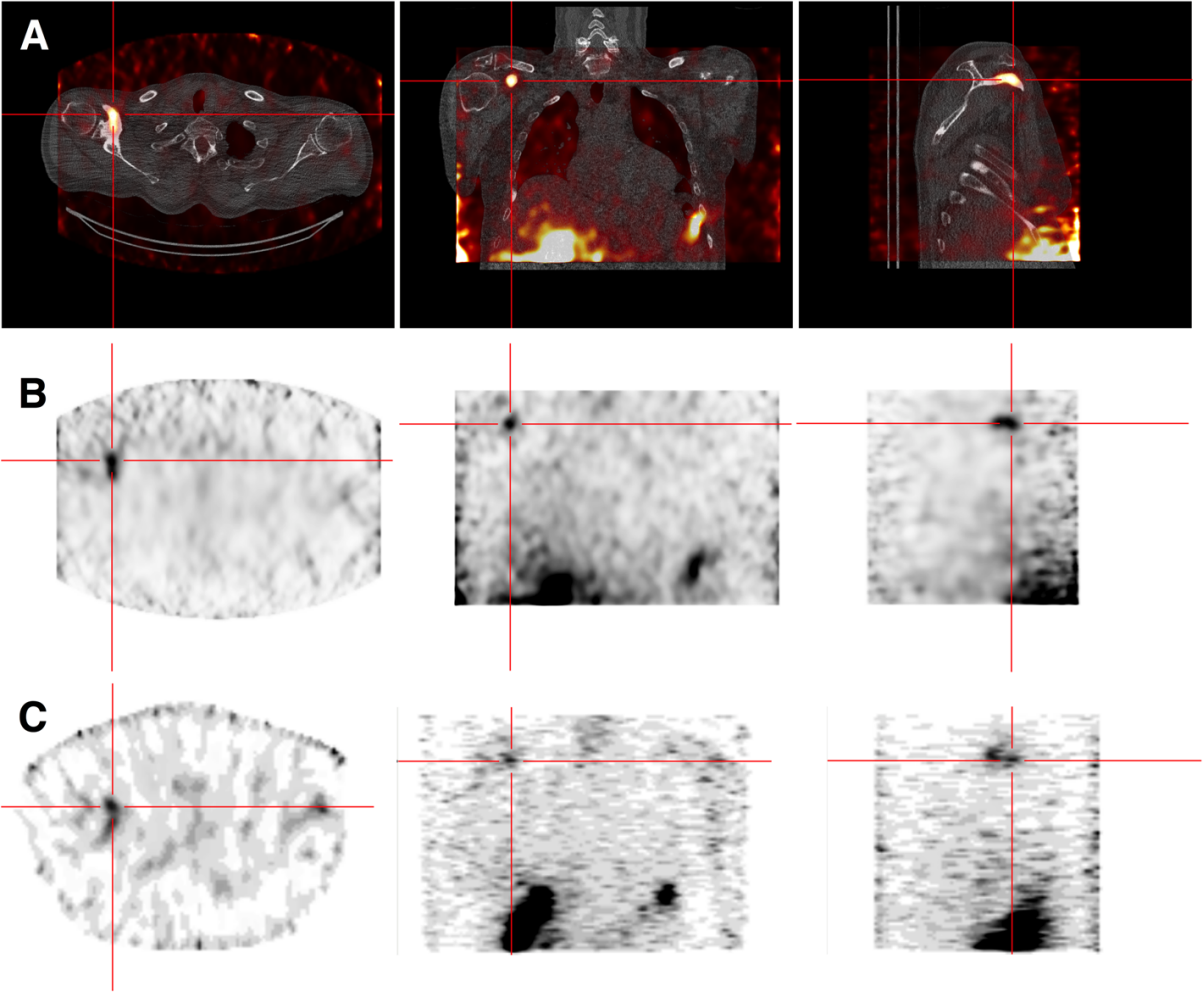
SPECT imaging of 30-min duration performed successively on CzT (with (**a**) and without (**b**) CT image fusion) and Anger cameras (**c**) 48-h after intravenous injection of 5.27 MBq of ^223^Ra. An uptake on a right shoulder bone metastasis was visible on axial, coronal and sagittal slices (left to right)

An example of VOI manually drawn for CNR calculation is presented in Fig. 5, with results obtained for each patient on both cameras. The CNR obtained was 2 to 6 times higher for the CzT camera, depending on the patient case. Enhanced detectability for cold or hot lesions can be seen on the thyroid scan displaying a cold nodule and on a ^223^Ra focus in a blastic lesion in a prostate cancer patient (Fig. 6).

## Discussion

The originality of the camera tested in this study is due not only to the nature of the detector used, but also to the proposed architecture of the camera. Indeed, the arrangement of detectors in a ring configuration mimics a Positron Emission Tomography (PET) system configuration and gives access to only fully 3D images (i.e. planar images are not feasible). To evaluate the performance of this type of camera, the use of classic standards of measurement, such as those proposed by the NEMA, is not fully applicable because of impossibility of performing planar images and removing collimators. When it comes to the evaluation in tomographic mode, we decided to measure the global performance of the camera with parameters similar to those employed in clinical routine.

Spatial resolution depends both on intrinsic performances of the camera and on parameters used for image reconstruction. Iterative reconstruction algorithms used for image reconstruction are characteristic of each system, leading to inconsistent results when choosing the same reconstruction parameters to compare two different systems. Indeed, if the number of iterations used has an impact on the measured spatial resolution, the convergence of the algorithms is not the same. The choice of reconstruction parameters to be used must therefore be adapted for each camera. To obtain more realistic measurements and to fully evaluate spatial resolution of the systems, we tested system capabilities both in extrinsic mode and for phantoms representative of clinical conditions, i.e. for head and body phantom in presence of background noise, with the acquisition and reconstruction parameters used in clinical routine. For both systems, the actual extrinsic resolution was close to the pixel size which is 2.40 and 2.56 mm for the Anger camera and the CzT camera, respectively. We also found that the reconstruction algorithm used with the CZT camera converges less quickly, according to the number of iterations, than for the Anger camera. An increase in the number of iterations for the CzT camera could have further improved the estimated extrinsic spatial resolution. Moreover, we also noticed for the Anger camera that for a high number of iterations, when the resolution becomes close to the pixel size, artefacts began to be visible on line sources. When it comes to tomographic resolution, no significant difference was observed between the systems. The differences between the two cameras for head and body phantoms are mainly due to source position in the FOV and also to the acquisition and reconstruction parameters used, such as the matrix size, the number of iterations or the reconstruction filters applied.

The energy resolution in this study was determined for a set of radioelements currently used in nuclear medicine. As it is impossible to remove collimators on the CzT camera, these were kept in place on both cameras to perform spectral measurements. This had an impact on measurement accuracy, due to the scattered photons created from interactions in the collimator material. In particular, we saw that the standard deviation was relatively higher for the lowest peak of dual energy isotopes (for example, at 70 keV for ^201^Tl) or for peaks with a relatively low count rate intensity (at 167 keV for ^201^Tl or at 171 keV for ^111^In). Despite this quantitative bias of measurement, the results obtained clearly showed the superiority of solid-state detectors over NaI(Tl) scintillation crystals, which corroborates the results obtained in previous studies comparing dedicated cardiac CzT-based camera with Anger cameras from various suppliers [1–3]. As a result, CzT detector can better discriminate the photoelectric peaks of two isotopes with a close photon energy emission, such as ^99m^Tc and ^123^I. The introduction of a multipurpose CzT based camera offers the possibility to use two isotopes simultaneously and may yield new indications [8].

Sensitivity comparison of two systems based on different architecture is difficult, as results depend closely on measuring conditions. To avoid conditions that would favour one of the two systems, sensitivity was estimated for different measurement settings and source geometries. This study showed a higher sensitivity of the CzT camera, whatever the source geometry, and with or without the use of the focus mode. This can be explained by the nature of the detector used, CzT detectors having the particularity to be more sensitive than NaI detectors. Nevertheless, we saw that the global sensitivity was much higher when the focus mode was activated for point source imaging, whether in air or at the centre of the head and body phantoms. This can be explained by the swipe motion of the detectors during acquisition. In the case where the source is much smaller than the size of the field of view, photon counts can only be recorded during the period when the source is facing the detector’s input surface. In order to benefit from the high sensitivity of CzT detectors, it is therefore important to use the focus mode to constrain the movement of the detectors to the region to be explored. This will be particularly the case for imaging small organs such as the heart, thyroid or kidneys.

Image quality evaluation using a Jaszczak phantom showed a superior image resolution with more visible rod sections and a significant gain in contrast visible in the cold spheres, as opposed to results in terms of spatial resolution discussed above. Indeed, while spatial resolution measured with point sources did not show any significant difference between the two cameras (Fig. 2), the visual assessment of the resolution with cold capillaries of the Jaszczak phantom showed a better resolution on the CzT camera, the smallest rods detectable by the CzT system being 6.4 mm versus 9.5 mm for the Anger gamma camera, as shown in Fig. 3. Detection of small rods are not only explained by intrinsic performance of the camera, but also by the proprietary OSEM algorithm used for image reconstruction. Indeed, the proprietary OSEM algorithm used on the CzT camera includes a pre-iteration convolution kernel allowing noise suppression and improvement of image quality. In addition, we have seen that the decrease in acquisition time preserved global image quality for the CzT camera, with in particular, a much lower noise level under conditions of low count statistics compared to the Anger camera. The gain in sensitivity combined with better image contrast, uniformity and noise level made it possible to reduce acquisition time by a factor of about 2 or 3 to obtain an image quality similar to that obtained with the Anger camera.

Clinical images obtained for a set of clinical applications and a variety of radiopharmaceuticals, ranging from the widely used ^99m^Tc to the biodistribution of ^223^Ra, in line with phantom measurements, demonstrated the superiority of the CzT camera in terms of image quality, with a gain in image contrast compared to the Anger camera when the same acquisition time was used. The impact of acquisition time reduction on image quality has not yet been assessed in clinical conditions, which can be regarded as a limitation of the present study. Further studies are ongoing to demonstrate that a gain in sensitivity makes possible the decrease of acquisition time and/or activity administered to the patient, as demonstrated by our phantom measurements. This may drastically change patient management. In particular, scan time reduction may allow the direct use of 3D imaging instead of planar imaging or extend the exploration length without increasing the duration of the examination. Additionally, as shown in Fig. 6, the gain in sensitivity is expected to be useful in situations where a low count statistic is faced, such as the biodistribution imaging of ^223^Ra. Moreover, we have seen that the focus mode considerably improved count performance. Therefore, the use of this mode will optimize the imaging of small organs, such as the heart or thyroid, as shown in Fig. 5.

## Conclusion

Measurements performed in this study to compare the performance of the newly available 3D SPECT CzT based system to a conventional Anger camera showed a better sensitivity, energy resolution and image contrast. The increased sensitivity observed using the focus mode paves the way to a significant reduction in acquisition time and total activity injected into patients for medium or small size organs. Given the impact that dedicated cardiac CzT has had on patient management in nuclear cardiology, the availability of a multipurpose CzT camera could impact all conventional nuclear medicine practices in the same way.

## Data Availability

Data are available upon reasonable request.

## Abbreviations

CNR: Contrast to noise ratio
Cpp: counts per pixel
CzT: Cadmium zinc telluride
DMSA: Dimercaptosuccinic acid
FWHM: Full width at half maximum
GE: General electric
HMDP: Hydroxymethylene diphosphonate
IEC: International Electrotechnical Commission
NaI(Tl): Sodium iodide doped with thallium
NEMA: National Electrical Manufacturers Association
OSEM: Ordered subset expectation maximization
PET: Positron emission tomography
RMS noise: Root mean square noise
ROI: Region of interest
SPECT: Single photon emission computed tomography
